# Dynamic Assessment of Reading Difficulties: Predictive and Incremental Validity on Attitude toward Reading and the Use of Dialogue/Participation Strategies in Classroom Activities

**DOI:** 10.3389/fpsyg.2017.00173

**Published:** 2017-02-13

**Authors:** Juan-José Navarro, Laura Lara

**Affiliations:** ^1^Instituto de Estudios en Ciencias de la Educación, Universidad Autónoma de ChileSantiago, Chile; ^2^Individual Differences, Language and Cognition Lab, Department of Developmental and Educational Psychology, University of SevilleSeville, Spain; ^3^Facultad de Psicología, Universidad Autónoma de ChileTalca, Chile

**Keywords:** dynamic assessment, personal-social adjustment, reading difficulties, intellectual disabilities, metacognition, incremental validity

## Abstract

Dynamic Assessment (DA) has been shown to have more predictive value than conventional tests for academic performance. However, in relation to reading difficulties, further research is needed to determine the predictive validity of DA for specific aspects of the different processes involved in reading and the differential validity of DA for different subgroups of students with an academic disadvantage. This paper analyzes the implementation of a DA device that evaluates processes involved in reading (EDPL) among 60 students with reading comprehension difficulties between 9 and 16 years of age, of whom 20 have intellectual disabilities, 24 have reading-related learning disabilities, and 16 have socio-cultural disadvantages. We specifically analyze the predictive validity of the EDPL device over attitude toward reading, and the use of dialogue/participation strategies in reading activities in the classroom during the implementation stage. We also analyze if the EDPL device provides additional information to that obtained with a conventionally applied personal-social adjustment scale (APSL). Results showed that dynamic scores, obtained from the implementation of the EDPL device, significantly predict the studied variables. Moreover, dynamic scores showed a significant incremental validity in relation to predictions based on an APSL scale. In relation to differential validity, the results indicated the superior predictive validity for DA for students with intellectual disabilities and reading disabilities than for students with socio-cultural disadvantages. Furthermore, the role of metacognition and its relation to the processes of personal-social adjustment in explaining the results is discussed.

## Introduction

Various models of Dynamic Assessment (DA) have been developed based on Vygotsky's pioneering studies on the social construction of concepts in childhood and intervention in the *zone of proximal development* (Vygotsky, 1934/1995, pp. 179–181), as well as on Feuerstein's research on *mediated learning experience* and *structural cognitive modifiability* (Feuerstein, 1996). Despite the differences between them, all of these models aim to optimize the assessment process, seeking information about the learning potential of students and their need for support during the mediation process (Sternberg and Grigorenko, 2002; Haywood and Lidz, 2007; Lidz, 2014). In this regard, DA may be defined as an assessment procedure focused on intervention that seeks to supplement the information that can be obtained through static or conventional tests, allowing for the generation of data beyond the final outcome of the activity. Thus, the learning process assessment that occurs during the activity is one of the goals of DA models, along with the determination of the extent to which students benefit from the assistance provided (Resing, 2000; Saldaña, 2004; Dörfler et al., 2009; Gustafson et al., 2014).

Since its inception, most DA procedures have been developed within the context of students with intellectual disabilities, learning difficulties, or socio-cultural disadvantages, and these are precisely the students for whom the level of effectiveness in the implementation of DA seems to be greatest (Campione and Brown, 1987; Carlson and Wiedl, 2000, 2013; Saldaña et al., 2007). In this regard, several studies have shown that children with very different schooling histories could achieve similar results on tests that assess their learning potential (Kaniel et al., 1991; Calero et al., 2013). There is also evidence of significant improvements in cognitive performance among students with special needs when they participate in assisted or collaborative learning situations, as well as following participation in DA processes. These studies reveal significant effects in the treatment groups (Feuerstein et al., 1979; Guthke and Beckmann, 2000; Saldaña et al., 2007; Beckmann et al., 2009; Resing et al., 2009; Resing and Elliott, 2011). Furthermore, studies also show significant gains in relation to specific domains such as reading or arithmetic (Swanson and Howard, 2005).

Moreover, it is well known that children with cognitive deficits, socio-cultural disadvantages, or learning difficulties obtain low or very low scores on standardized tests. However, these tests do not provide information about student's learning processes, or about how we could help these students to improve their performances. Therefore, DA would offer a fairer assessment of students with disadvantages, either social, cultural, or cognitive, as it assesses the development of learning potential, adjusting for the student's initial level. Various studies have shown that the assessment of students' competence provides greater and richer information in situations where they participate in mediated learning activities (Swanson and Howard, 2005; Caffrey et al., 2008). These activities would provide the best way to approach those processes that are not yet manifested by the student, but could be with the necessary help.

This additional information can also relate to the possibility of obtaining indicators of the incremental validity of dynamic models for various aspects of performance in relation to static tests (Beckmann, 2006). Studies have shown the additional predictive validity of dynamic tests (Calero, 2004; Caffrey et al., 2008), especially when the prediction has taken place in relation to learning situations that were conceptually and methodologically in line with the DA contents and procedures (Jensen, 2000; Sternberg and Grigorenko, 2002; Saldaña et al., 2007; Navarro and Mora, 2013). Thus, the relationship between the content of both processes, assessment and educational intervention, would be based on the highest rates of predictive validity of DA in the school context.

Research on DA has primarily focused on analyzing general cognitive functions, such as intelligence, and finding measures that better predict subjects' subsequent academic performance relative to IQ. To a lesser extent, studies have focused on the effect of the contextualized implementation of DA models in relation to specific domains of school learning or in relation to processes of personal-social adjustment, such as motivation or attitudes (Kozulin and Garb, 2002; Sternberg and Grigorenko, 2002). However, although statistical indicators of validity and reliability have been obtained for some dynamic tests (Caffrey et al., 2008), these decontextualized approaches based on general cognitive functions do not seem to have led to improvement in the learning behaviors of the students or in assessment processes in the school setting (Delclos et al., 1992; Ruijssenaars et al., 1993; Jensen, 2000; Elliott, 2003). Although some studies demonstrate the predictive power of these DA tests for academic performance, most of these studies focus on dynamic approaches that emphasize the decontextualization of tasks and the evaluation process (Lauchlan and Elliott, 2001; Sternberg and Grigorenko, 2002). In this sense, poor connections between the activities and contents of the evaluation processes and educational interventions could significantly affect the ecological validity of the obtained conclusions (Elliott, 2003; Thurman and McGrath, 2008).

### Dynamic assessment of reading competence

In recent years, there has been a rediscovery of the application of DA procedures to specific domains of school learning, contributing to a revision of previous contributions in the field with regard to the teaching-learning process (Guterman, 2002; Kalyuga and Sweller, 2005; Swanson and Howard, 2005; Haywood and Lidz, 2007; Thurman and McGrath, 2008; Fuchs et al., 2011; Resing and Elliott, 2011). This renewed interest in DA corresponds to changes in the conceptualization of reading processes as well as in the treatment of learning difficulties. In this regard, considering systemic and interactive educational approaches is particularly important. These approaches emphasize the constructive and metacognitive activity of the reader (Thiede et al., 2003; Hacker et al., 2009). Consequently, these approaches highlight the importance of acting on the competencies of students to control and regulate the reading process, to interpret texts in a progressively autonomous way, to question the content of texts, to plan and monitor the achievement of objectives, and to evaluate their own understanding.

DA models' preferential consideration of these and other processes involved in reading helps overcome barriers to establishing dynamic procedures in this area. These barriers concern the “crystallized” quality of specific domains such as reading (rendering it difficult to assess short-term modifiability) and the relevance of prior knowledge in the assessment of students' understanding (rendering it difficult to establish control over students' learning history; Kozulin and Garb, 2002). As researchers have focused more on reading processes than on textual content and comprehension as a product, the consideration of assessment models focused on the students' response to educational intervention and on the learning process has become more important.

### Predictive validity of dynamic assessment for reading difficulties

Revision of previous contributions of dynamic decontextualized approaches also involves a rethinking of research on the predictive validity of dynamic models. In this sense, some studies have aimed to determine the predictive power of dynamic applications for reading relative to static testing. For example, Hamers et al. (1994) have found that students' reading results were more highly correlated with the dynamic application of a *learning test* that included specific reading content than with an IQ test or a general, conventionally-applied *learning test*. On the other hand, in a study conducted with students with disabilities, Lidz et al. (1997) also found that dynamic applications of specific subtests of the *Cognitive Assessment System* based on the PASS model (Naglieri and Das, 1987) showed significant improvements in predictive validity for reading compared with static testing. Meanwhile, in a longitudinal analysis of the reading achievement of a mixed-ability group of children from kindergarten to fifth grade, Byrne et al. (2000) found that, based on multiple regression models, the dynamic score that reflected the rate of progress in reading over several weeks predicted academic performance significantly better than the static post-test score. Data further showed a validity of between 9 and 21% for the different performance measures. Resing (1993) also demonstrated the incremental validity of dynamic measures for primary students with disabilities in comparison with static tests. In this sense, after controlling for verbal IQ, the dynamic scores predicted an additional 13% of the variance in verbal achievement measures, such as reading and writing, and 14% of the variance in teacher ratings of school performance.

In general, studies that have examined the additional predictive validity of DA for academic performance in specific domains have included as potential predictors, first, the pre- and post-test measures of conventional reading tests and intelligence tests, and second, the scores from various dynamic tests. It is also noteworthy that studies have found that the predictive validity of academic performance for students with disabilities was more accurate than for students with a sociocultural disadvantage or with typical development (Caffrey et al., 2008).

However, in relation to reading and learning difficulties, further research is needed to determine the predictive value of DA models for more specific aspects related to the processes involved in reading (Carlson and Wiedl, 2000). Regarding reading, various researchers have recently highlighted the relevance of personal-social adjustment processes related to the socio-educational learning context and other aspects related to motivation, self-efficacy, and attitude (Guthrie et al., 2000, 2007; Meltzer et al., 2004; Taboada et al., 2009). Similarly, researchers' interest in DA has diversified, extending to the intervention and measurement of change in various specific aspects that go beyond IQ, such as self-regulation skills and personal-social adjustment at school (Kozulin, 2014; Lidz, 2014). Thus, to account for specific domains such as reading in DA procedures, we must consider the different processes involved in the domains studied.

Regarding the relevance of these socio-personal adjustment processes in reading, an increasing number of studies have attempted to determine, among other things, how such processes influence reading development, what other factors are involved, what role each process plays, what relations exist between processes involved in reading, and how these processes affect students with learning difficulties or disabilities. Thus, along with more commonly studied learning difficulties related to word recognition and textual integration processes, other problems have been described that also characterize students with reading difficulties. The most common difficulties are related with socio-emotional aspects, identity development, self-esteem, frustration tolerance, attributional patterns of success or failure, and reading disengagement (Borkowski et al., 1986; Greshman and MacMillan, 1997; Fulmer and Frijters, 2011; Guthrie et al., 2012). In addition, the interconnection between several of these problems should be noted. Thus, feelings of frustration, inability, or failure among certain students are often attached to a devalued perception of themselves, their abilities, and their academic possibilities. Additionally, a possible effect of mutual reinforcement arises between students' low expectations and teachers' negative perceptions (Meltzer et al., 2004; Jussim and Harber, 2005; Hornstra et al., 2010). Such an effect usually results in a negative attitude toward reading and learning activities for students. Given the importance of these aspects for educational and socio-personal development along with their complex interdependency, any reading assessment process should include these types of factors by analyzing their influence (Mora-Merchán and Mora, 2000). Furthermore, considering the context of the classroom, it is particularly relevant both for assessing the difficulties associated with reading and for identifying the conditions that favor the development of these adjustment processes (e.g., groupings, the most suitable type of support, ways of teaching reading, and the development of specific abilities).

Considering attitudes toward reading and dialogue/participation strategies as *forms of developing expertise* (Sternberg and Grigorenko, 2002) and accounting for the interactive nature of DA procedures could provide DA models with greater access to contextualized information about personal-social adjustment processes involved in reading. Such information may be related to reading difficulties that occur in school-related activities. Research reveals the essential role of affective-motivational factors related to school engagement and strategic activity for the development of cognitive tasks (Hessels-Schlatter, 2010; Guthrie et al., 2012). Indeed, students should assess the benefits of implementing the strategies that have been previously taught and practiced, evaluate whether they feel competent in using these strategies, perceive whether they can control and regulate the use of these strategies, and be motivated to employ them. In this sense, one of the arguments in favor of DA models is their ability to provide a more integrated assessment of the various processes and skills that are involved in an activity (Rapp and Broek, 2005), as well as their greater validity in determining the learning potential and skill development of students with socio-cultural disadvantages, students with disabilities, and ethnic minorities—aspects that might otherwise be hidden (Dörfler et al., 2009; Resing et al., 2009). DA models emphasize the evaluation of students' learning processes and learning abilities, focusing primarily on skills that are *in development* and thus allowing for the retrieval of information that eludes conventional aptitude tests (Sternberg and Grigorenko, 2002; Dörfler et al., 2009). This information should be qualitatively different from and additional to that which conventional reading tests can provide (Carlson and Wiedl, 2013).

### The present study: objectives and hypotheses

The purpose of the present study is to analyze the extent to which the implementation of a DA device offers a significant increment in the predictive validity (incremental validity) of attitude toward reading and the use of dialogue/participation strategies in classroom reading activities in relation to the prediction based on the conventionally administrated Personal-Social Adjustment (APSL) scale. With this aim, this paper analyzes the implementation of a DA device that evaluates processes involved in reading [EDPL in (Country, Authors)] among three subgroups of students with reading difficulties: an intellectual disability (ID) subgroup, a learning disabilities (LD) subgroup, and a socio-cultural disadvantages (SCD) subgroup. The predictive validity of the attitude toward reading and the use of dialogue/participation strategies in classroom reading tasks were examined. Additionally, we analyzed the extent to which the EDPL device offers additional information to that obtained with the APSL scale. We expected that the dynamic scores obtained from the contextualized application of the EDPL device would be significantly correlated with students' attitude toward reading and the use of dialogue/participation strategies in classroom reading activities, and these correlations would be greater than for the APSL scale (Hypothesis 1). Furthermore, we expected the dynamic scores to constitute an additional explanatory factor (incremental validity) for students' attitude toward reading and the use of dialogue/participation strategies in classroom reading activities in relation to the prediction based on the APSL scale (Hypothesis 2). Finally, we expected to find differences in the incremental validity indexes for each of the three subgroups studied. In this sense, and in line with previous studies (Caffrey et al., 2008), we expected to find greater incremental validity indexes for the dynamic scores of the ID subgroup relative to the other subgroups (Hypothesis 3). This third hypothesis is based on the special relevance that could have specific aspects such as motivation, attitude, self-concept, expectations or patterns of causal attribution, in the learning process of socio-culturally disadvantaged students, as well as the interaction of these aspects with the educational guidelines in the school or in the family, the expectations and patterns of causal attribution of teachers and parents, and the difficulties related to the specific school context.

## Materials and methods

### Participants

Sixty students from Seville and Cadiz (Andalusia-Spain) with reading difficulties (32 from primary school, 20 boys and 12 girls; 28 from secondary school, 17 boys and 11 girls) between 9 and 16 years of age (*M* = 12.40, *SD* = 2.44) participated in the study. Twenty-four of them were classified as students with LD and without ID or SCD; 16 were classified as students with SCD and without ID, and 20 were classified as students with ID (*IQ* = 75). The selection of the initial sample and the allocation of students to the different subgroups was based on information about the significant presence of reading difficulties provided by the specialist teachers/counselors in a data-collection form along with the IQ scores obtained on the students' pre-tests (cognitive performance was evaluated with Cattell's Factor “G” test, scale 2; Cattell and Cattell, 1974/2001). Likewise, the operationalization of the socio-cultural disadvantages was made with the information provides by the institutions about socio-economical and socio-cultural level of the student's family (educational level, availability of communitarian services, etc.). The study presented is part of a broader investigation (Navarro and Mora, 2011, 2012) involving 13 educational centers, 7 primary schools, and 6 secondary schools. The entire sample included an experimental group where the EDPL device was applied (*N* = 60) and two control groups (control group: with reading difficulties, *N* = 73, and control group 2: without difficulties, *N* = 202). The socio-economic level of the sample group was classified as middle-low based on the information provided by the institutions. The students were assigned to the experimental group or control group depending on the participation possibilities of the teachers who collaborated in the study. Twelve support teachers/counselors formed the working group that implemented the EDPL device to the experimental group, and another five support teachers/counselors were in the control group. In both cases, these individuals were professionals involved in innovative projects with training and at least 5 years of experience in special education, psychology, or pedagogy.

### Design and procedure

The research framework for this study (Navarro and Mora, 2012) used a pre-post Quasi-experimental design with a control group. Its main objective was to analyze the experimental application of the EDPL device in the school context. Specifically, the study aimed to assess its effectiveness regarding the information that could be obtained with its application; examine its predictive validity in relation to performance and progress in reading; set key mediation guidelines for optimizing the teaching-learning process that is linked to the implementation of contextualized reading-specific activities; and assess its potential to induce changes in the learning process of students with reading difficulties. This research framework also envisaged a process of continuous evaluation in which it is possible to obtain data regularly, which allows us to evaluate the learning process. In accordance with this study's objectives, in this paper we focus on the experimental group to analyze the predictive validity of the EDPL device on the personal-social adjustment processes involved in reading. In previous studies we have analyzed the predictive validity of the EDPL device on reading performance and progress (Navarro and Mora, 2013), as well as the effect of its implementation on the observed improvements in reader performance, intelligence and personal-social adjustment (Navarro and Mora, 2012). These studies showed significant gains for the experimental group and not for the control group, especially when the students had scored significantly lower in reading comprehension and also in general intelligence. The main interest of this study, and what constitutes its essential contribution, is to determine the extent to which the EDPL device offers additional information on processes of social and social adjustment in relation to a conventional evaluation procedure.

The experimental implementation of the EDPL device occurred over 16 weeks, and it was performed by 12 support teachers/counselors (evaluators) who had training and experience in the field of special education, and who also received specific training on the theoretical/methodological basis of the proposal. The EDPL device was implemented in the school settings of the students and in small groups (between 3 and 8 participants). Each of the 11 groups had an average of 2 weekly sessions of 45–50 min. These conditions were equivalents for the control group because this group was also made up of students with reading difficulties who received different programs of academic support in small groups depending on the diversity of educational centers and participant teachers.

The predictive value analysis was performed on the basis of the Classroom teachers' assessments of the performance observed during the implementation phase (Resing, 2000; Caffrey et al., 2008). The classroom teachers did not participate in the implementation of the device and did not have knowledge of the distribution of the students in relation to their participation in the experimental or control group. In this sense, teacher assessment as a measure of student performance potentially gives greater ecological validity to the study, allowing for the evaluation of procedural elements that cannot be measured with static performance measures. Also, teacher's assessments of students have proven to be an excellent predictor of academic performance (Clemente et al., 1999). We developed a template/record in which the faculty, once the implementation was completed, rated students' performances using a 4-point Likert scale (1 = *Low-very low level*, 2 = *Middle-low level*, 3 = *Middle-high level, and* 4 = *High-very high level*) for seven evaluation criteria related to academic performance in reading. Two of these evaluation criteria were related to personal-social adjustment processes: “*Uses adequate strategies in dialogue situations in the classroom: listening, respecting opinions, expressing their points of view*” and “*Displays a positive attitude toward reading*.” The inclusion of these evaluation criteria in the registration-evaluation sheet was due to the fact that they were considered to be especially relevant for the assessment of the performance and progress of the students who participated in the study. Their consideration in the present study will allow us to establish the predictive validity of the EDPL device on the scores given by the faculty in relation to these processes.

Regarding ethical considerations, when the study was performed there was no formal ethics committee at our institutions. However, the research project was approved by the Department of Developmental and Educational Psychology at the University of Seville, and it was carried out in accordance with the ethical considerations of the Declaration of Helsinki. All the participants were informed about the aims of the study and gave informed consent. Permission from the directors of the school centers, teachers and families were requested in personal interviews and meetings with those involved. Additionally, prior to asking for participation, students were informed of the aims of the study.

### Instruments

#### The EDPL device

The EDPL device (Navarro and Mora, 2012) consists of 32 evaluation/intervention activities structured through networks and in line with the reading processes that were considered based on previous research (Graesser et al., 1994; Hacker, 1998; Kintsch and Kintsch, 2005; Compton et al., 2010). We grouped these processes into three blocks: (1) *Analysis and Integration of Information*, including (a) underlying psychological processes (memory, attention, and visual perception), (b) grapheme-phoneme association, (c) textual integration processes, and (d) text-knowledge integration processes; (2) *Metacognitive Processes*, including (a) meta-knowledge related to reading, strategies, content, and affective-motivational aspects, and (b) self-regulation processes of comprehension; and finally, (3) *Personal/Social Adjustment Processes* (Table 1). Based on the proposed objectives and the design of the framework research of the present study, the evaluators developed the different activities of the EDPL device following the sequence outlined in the mentioned table. Likewise, each of the activities of the EDPL device includes (a) the process to be evaluated, (b) a description of the task, (c) the methodology of implementation, (d) mediation guidelines, and evaluation of metacognitive processes, and (e) a set of assessment indicators related to the different processes.

**Table 1:** **Blocks of reading processes and activities included in EDPL device**.

**Analysis and integration of information**
Underlying psychological processes 1 Visual Continuation/1 2 Visual Continuation/2 3 Perceptual discrimination/1 4 Perceptual discrimination/2 5 Perceptual discrimination/3 6 Catch up with the same words 7 Word composition 8 Temporal Sequence 9 Working memory and memory strategies
Grapheme-phoneme association 10. Reading pseudo-words — — — — — — — - *11. Assessment of the phonological awareness* *12. Phonological decoding and Grapheme-Phoneme association*
Text Integration Processes 13. Reading of words — — — — — — — - *14. Reading of homophones and homophones* *15. Incorporation of words into the internal visual lexicon* — — — — — — — — - 16. To put the sentences into the right order 17. To relate sentences and drawings 18. To elaborate new sentences — — — — — — — - *19. To complete sentences with the missing Word* *20. Oral sentence construction* — — — — — — — - 21. Puntuation marks 22. Use of the text structure 23. Meaning extraction and construction
Text-Knowledge Integration Processes 24. Elaboration of hypotheses 25. Integration and Inferences 26. To complete the end of the text 27. Previous Knowledge 28. Relation construction
**Metacognitive processes**
Meta-Knowledge and Self-regulation 29. To compare two texts 30. Linguistic incoherence 31. How can we understand better?
**Personal-social adjustment processes**
32. Assessment of the context

*Activities in italics, which are under the dashed lines, were performed in the event of significant difficulties in the previous activity or group of activities*.

To facilitate the collection and assessment of the mediation process, evaluators had *registration-evaluation sheets* for each activity. Evaluators used these sheets to assess the implementation of the assessment indicators that are collected at the end of each activity. These assessment indicators were related to the reading processes to be evaluated in each of the activities. Thus, the assessment of learning process takes place on the EDPL device through the evaluation of these assessment indicators, along with proposed mediation guidelines. These are the components that allow us to obtain information about the difficulties experienced by the students during the process, as well as the type and degree of support required for its optimization, making connections between assessment and intervention (Dörfler et al., 2009; Resing, 2013). The aim is to obtain information on the contextualized learning potential, establishing not only the difficulties of the students, but also, and especially, the mediation guidelines that could facilitate the optimal execution of the task or the procurement of valuable information oriented to intervention. In this sense, the mediation process proposed in the EDPL device includes a set of methodological guidelines (not standardized) as a metacognitive guide, graduated prompts, questions, or feedback, which is intended specifically for each of the activities. Evaluators should apply these guidelines in order to evaluate both the implementation process and the support needed by the students during the resolution of tasks, as well as to observe to what extent students incorporate some of the strategies used in the mediation process.

The total number of assessment indicators was 149, of which 60 were related to the first block of processes, *Analysis and Integration of Information* (13 indicators were related to basic psychological processes, four to the processes of grapheme-phoneme association, 25 to textual integration processes, and 18 to textual integration processes of prior knowledge), 71 indicators were related to the second block, *Metacognitive Processes*, and 18 were related to the third block, *Personal-Social Adjustment Processes* (Table 2). To quantify the evaluators' DA of the indicators proposed in the EDPL device for each process listed, we developed a system of analysis that allowed us to operationalize the implementation process and to obtain dynamic scores. Thus, the global dynamic scores obtained come from an integrated assessment of the different processes covered by the EDPL device following the assessment of the indicators in each activity. The adopted system (Moreno and Saldaña, 2005) was based on a graduated scale comprising four points: 1 (*Behavior is not shown, or the indicator has not been put into practice)*, 2 (*Signs of behavior or a rudimentary application of the indicator*), 3 (*Clearly observable behavior, although not of great quality*), and 4 (*Intense or high-quality behavior*).

**Table 2:** **Assessment indicators involved in personal-social adjustment processes related to reading that were covered in the various activities of the EDPL device**.

**Motivation, attitudes, and interest in relation to reading**
Has intrinsic objectives focused on reading competency.
Persists when facing obstacles or difficulties.
Chooses challenging tasks beyond the limit of his/her current abilities.
Shows enthusiasm for reading/shows interest during activities related to reading.
Shows pride and self-confidence as a reader.
Believes that he/she can improve his/her own reading/writing. Takes an active role.
Believes that others respect his/her contributions.
Corrects his/her own mistakes without showing aggression or depression. Does not manifest anxiety or fear of failure.
Maintains his/her own opinions when warranted. Does not give in to peer pressure, and counteracts against suggestions.
Selects voluntary reading and writing as free-choice activities.
**Cooperation with classmates in reading tasks**
Frequently collaborates in reading activities.
Starts or actively participates in discussions, dialogues, or debates on the meaning of texts.
Provides positive support, affection, and educational support to his/her peers.
Performs a variety of roles in the learning community.
Values the contributions of others and respects their opinion and help.
Emergence/Request of assistance/collaboration behaviors.
**Connecting reading with the curriculum**
Contemplates reading, writing, speaking and listening as mutually supportive activities.
Understands that what he/she learns in reading and writing is useful in other subjects.

#### The personal-social adjustment (APSL) scale

Furthermore, the APSL scale (Navarro and Mora, 2012) was among the various test criteria used to assess the predictive and incremental validity of the EDPL device and its effect on the experimental subjects. The scale assesses personal-social adjustment processes related to reading and comprises 80 items grouped into nine dimensions that are presented to the students in short sentences with which they individually express their agreement or disagreement (for example: *I like reading a lot, although it costs me; I am unable to understand most texts; When I make mistakes by reading or when I do not understand something, I usually give up the task; My family usually helps me a lot with reading when I need it*). The maximum obtainable score is 80 points. To control for possible reading comprehension difficulties, the items are read out loud twice by the evaluator. A pilot study was conducted to observe the various aspects related to the assessment's validity and reliability along with the estimated execution time, the difficulty or ease of the proposed items, the comprehensibility of the instructions provided, as well as the interest, attitude, and motivation with which the students completed the test. The analysis and expert validation was performed prior to the implementation and yielded positive results. The assessment was implemented with 5th and 6th grade primary education students and 2nd and 4th grade secondary education students (*N* = 98) from two educational centers. Among other data, the following were obtained: *M* = 55.49; *SD* = 10.77; Cronbach's alpha for internal consistency = 0.88; Kolmogorov-Smirnov normality test = 0.077 (*p* = 0.179); average homogeneity index = 0.32; and average items value = 0.69. Likewise, the initial equivalence of the experimental group and the control group in the pre-test scores was analyzed, including the presence of students from the three subgroups. No significant differences between the groups were found in the analyses (Navarro and Mora, 2012). The values of Levene's test of variance homogeneity also showed this initial equivalence. The groups were also equivalent with regard to age and the sex.

### Data analysis

Analyses were performed using SPSS/PC-20 statistical program. To analyze the additional predictive value of the EDPL device, four separate hierarchical multiple regression analyses were performed, one for the entire group and another for each of the different subgroups studied. First, for each contrast in the first block, we entered the post-test measures for the personal-social adjustment (APSL) scale, and then we entered the dynamic score as an additional predictive value. The main dynamic measure was the overall score obtained by the EDPL (DS-EDPL). This measure should provide additional meaning for the prediction of students' attitudes toward reading, and the use of dialogue/participation strategies in relation to information obtained by the APSL scale. We also considered important to use a more specific measure of the dynamic scores obtained though the valuation of the indicators directly related with the personal-social adjustment processes (DS-PSA). The DS-PSA indicators are conceptually related to the items included in the APSL scale, thus allowing the evaluation of predictive validity of the part of the device more direct related with the criteria measure. Pearson correlation analysis was performed previously to determine the degree of association between the variables included in the regression, as well as to establish the correlation level between the different measures. Regarding the treatment of missing data, the measurements obtained reflect the available data, and the contrasts were made by taking into account those students who performed the necessary tests. The reason for the lack of data in some measurements is because not all students attended class on the day that the tests were applied or because not all teachers conducted performance assessments.

## Results

### Data quality and the reliability of the EDPL device

**Figure 1:**
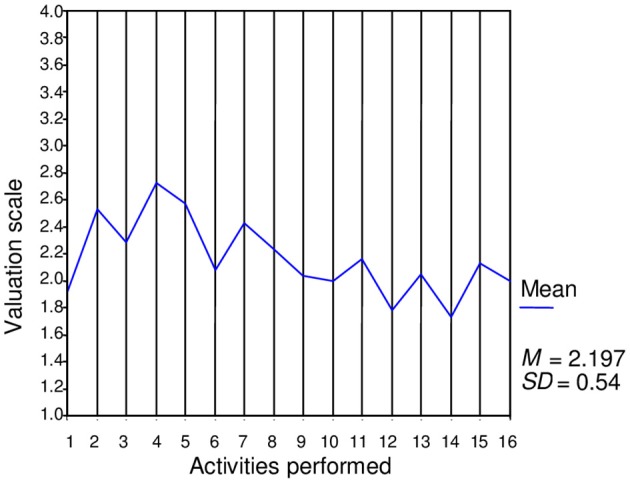
**Evolution of dynamic scores of personal-social adjustment processes in the 16 activities with specific indicators**.

**Figure 2:**
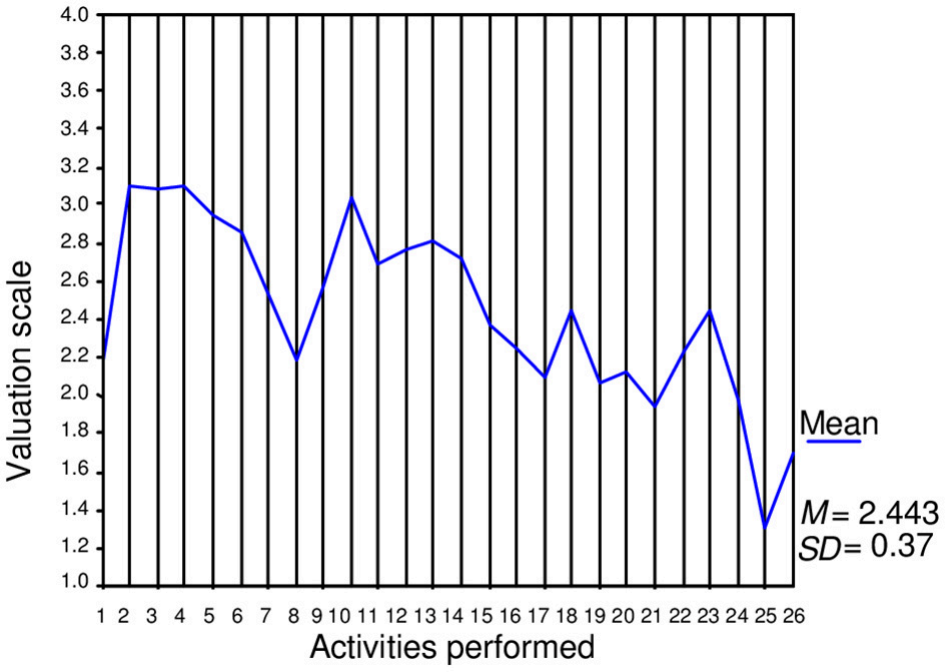
**Evolution of global dynamic scores through activities performed (26)**.

Figure 1 presents the average values of the dynamic scores obtained by the entire group for personal-social adjustment processes in each of the 16 activities performed, with indicators for these processes as well as the global average dynamic score for these processes. Figure 2 presents the average values of the global dynamic scores obtained by the entire group for each of the 26 activities performed as well as the mean global dynamic scores. The average values were calculated based on the highest obtainable direct score (four points). By calculating the coefficient of intra-class correlation, the analysis estimating inter-observer reliability provided evidence for the internal consistency of the ratings. For these calculations, we selected the activities that had the largest number of student participants. The activities selected were as follows: *Reading of pseudowords* (processes of grapheme-phoneme association); *Reading of words*; *To put the sentences into the right order*; *To relate sentences and drawings*; *To elaborate new sentences*; *Punctuation marks*; *Use of the text structure*; *Meaning extraction and construction (*textual integration processes); and *Integration and inferences (*text-knowledge integration processes). Analyses were performed on a total of 15 cases. The number of items (indicators assessed by the evaluators in the nine selected activities) was 149, and the total number of observations was 2235. The analysis revealed an alpha value of.98, an inter-item correlation of.26 (*F* = 7.46*, p* < 0.001), and an intra-class correlation coefficient of.29 for the simple measure (*F* = 60.81; *p* < 0.001 for 14 df).

The analysis of internal consistency of the nine selected activities showed an average alpha coefficient value of.83, with the lowest value being for *Reading of words* (0.70) and the highest for *Meaning extraction and construction* (0.94). With regard to construct validity, the correlation matrix between the scores on the various processes covered by the EDPL device and between these and the global dynamic scores reflects an average Pearson correlation coefficient value of.45 for the 21 contrasts made, with 14 significant contrasts with a probability level of *p* < 0.01 and 2 with a level of *p* < 0.05. The high number of significant correlations among (a) the various activities selected, (b) between the different processes considered in the EDPL device, as well as (c) between these processes and the global dynamic scores (Navarro and Mora, 2011), reinforces the consistency of the device in terms of content and the basic theory behind it, as well as the coding and assessment system used.

### Outcomes in the different measures and correlation matrices

In Table 3, we present the scores obtained for the entire group and each subgroup for the different measures. Table 4 reports the correlation matrix between these values and the scores for the entire group and for the ID subgroup. In Table 5, we present the matrix for the LD and SCD subgroups.

**Table 3:** **Descriptive statistics (averages and standard deviations) for the entire group and for the different subgroups considered**.

	**Entire group**			**ID Sub-group**			**LD Sub-group**			**SCD Sub-group**		
	**M**	***SD***	**N**	**M**	***SD***	**N**	**M**	***SD***	**N**	**M**	***SD***	**N**
Pre-test APSL	50.79	9.84	57	48.35	9.87	20	51.88	9.53	24	52.54	10.37	13
Pos-test APSL	50.63	10.27	57	48.61	10.71	18	51.24	10.40	25	52.14	9.82	14
IS APSL	0.10	11.55	54	0.55	9.75	18	−0.18	13.79	24	0.00	9.86	12
DS-PSA	2.20	0.54	60	2.16	0.53	20	2.30	0.60	25	2.07	0.45	15
DS-EDPL	2.44	0.37	60	2.40	0.34	20	2.53	0.41	25	2.37	0.32	15
DPS-CT	2.47	0.92	59	2.35	0.99	20	2.54	0.88	24	2.53	0.92	15
ATR-CT	2.54	0.93	59	2.60	0.99	20	2.38	0.82	24	2.73	1.03	15

*ID, intellectual disabilities; LD, specific learning disabilities; SCD, socio-cultural disadvantage; IS, improvement score; DS-PSA, dynamic scores obtained for personal-social adjustment processes; DS-EDPL, global dynamic score obtained for the EDPL device; DPS-CT, classroom teacher assessment regarding the use of dialogue/participation strategies in the classroom (0–4); ATR-CT, attitude (0–4) as assessed by classroom teachers*.

**Table 4:**
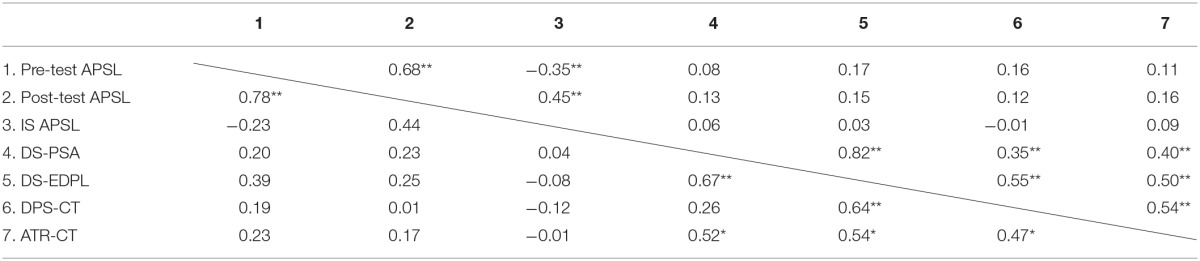
**Pearson correlation coefficients between the scores for attitude toward reading, and use of dialogue/participation strategies in the classroom for the entire group (over the diagonal) and the subgroup of students with intellectual disabilities (under the diagonal) and the scores obtained for the APSL scale and the EDPL device**.

IS, improvement score; DS-PSA, dynamic scores obtained for personal-social adjustment processes; DS-EDPL, global dynamic score obtained for the EDPL device; DPS-CT, classroom teacher assessment regarding the use of dialogue/participation strategies in the classroom (0–4); ATR-CT, attitude (0–4) as assessed by classroom teachers; * p < 0.05; ** p < 0.01 (Bilateral).

**Table 5:**
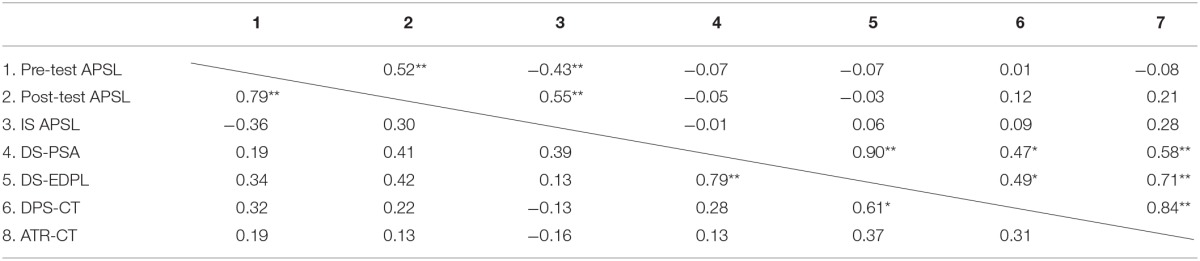
**Pearson correlation coefficients between the assessments regarding attitude toward reading, and use of dialogue/participation strategies in the classroom for the subgroup of students with specific learning difficulties (above the diagonal) and the subgroup of students with a socio-cultural disadvantage (under the diagonal) and the scores obtained for the APSL scale and the EDPL device**.

IS, improvement score; DS-PSA, dynamic scores obtained for personal-social adjustment processes; DS-EDPL, global dynamic score obtained for the EDPL device; DPS-CT, classroom teacher assessment regarding the use of dialogue/participation strategies in the classroom (0–4); ATR-CT, attitude (0–4) as assessed by classroom teachers; * p < 0.05; ** p < 0.01 (Bilateral).

As the data show, there was a high correlation between the global dynamic score for the EDPL device (denoted DS-EDPL in the results section and obtained from the average rating of the evaluators on a total of 149 indicators for the various processes contemplated) and the personal-social adjustment dynamic score (denoted DS-PSA in the results section and obtained only from the 18 specific indicators of this process). This correlation was obtained for the entire group (*r* = 0.82) and the different subgroups (*r* = 0.67 for the ID subgroup, *r* = 0.90 for the LD subgroup, and *r* = 0.79 for the SCD subgroup), demonstrating consistency between the assessment given by the evaluators relative to the device as a whole and to the assessment of the adjustment processes that are part of the device. Additionally, the high correlation between the DS-EDPL and the DS-PSA indicates the relevance of the implementation of skills related to the processes of social and personal adjustment in the performance of students on the EDPL device. Further, for the entire group, the DS-EDPL is significantly correlated with students' use of dialogue/participation strategies, and their attitudes toward reading as assessed by the classroom teachers. The DS-PSA is also significantly correlated with classroom teachers' assessments of the students' attitudes toward reading and the use of dialogue/participation strategies. For the ID subgroup, the DS-EDPL is significantly correlated with students' attitudes toward reading and their use of dialogue/participation strategies. For the LD and SCD sub-groups, the DS-EDPL is significantly correlated with students' use of dialogue/participation strategies. Moreover, the DS-PSA is significantly correlated with students' attitudes toward reading. Conversely, the measurements obtained in the APSL scale and the assessments given by teachers are not significantly correlated. In short, the data support our first hypothesis: the DS-EDPL and DS-PSA are significantly correlated with students' attitude toward reading, and the use of dialogue/participation strategies in the classroom, and the correlations are greater for the EDPL than for the APSL scale. Likewise, the DS-PSA was also correlated significantly with student performance evaluations (taking into account the seven evaluation criteria related to academic performance in reading) (*r* = 0.29; *p* < 0.05). On the other hand, the pre-test of the APSL scale obtained medium or low levels of correlations without statistical signification.

Although our study was focused on the implementation of the EDPL device on the experimental group, in order to provide a reference framework for interpretation of the results obtained we present now some comparative data including the control group. No significant group differences were found in the pre-test of the APSL scale as well as in the others tests-criteria used in our research framework (Navarro and Mora, 2011). In relation to the control group, only the pre-test score correlated significantly with classroom teachers assessments' of academic performance (*r* = 0.40; *p* < 0.05). Furthermore, the correlation level with academic performance as assessed by the classroom teachers was higher in the pre-test than the post-test. In this regard, the regression analysis performed for the control group showed that the only significant variable in the regression equation was the pre-test, with an *R*^2^ value of.21 (*F* = 11.13, *p* < 0.01). Meanwhile, in the experimental group equation, only the two dynamic scores were included, excluding the remaining model variables. First, it introduced the DS-EDPL with an *R*^2^ value of.31 (*F* = 22.76; *p* < 0.001), and secondly the DS-PSA, which caused an increase in the value of *R*^2^ of 0.08. These two variables explained 39.1% of the variance in performance by classroom teachers, resulting in a significant model (*F* = 16.05; *p* < 0.001).

### Incremental validity of the device

In order to test incremental validity of the device, hierarchical multiple regression analysis were run, for the entire group (Table 6) as well as by each of the subgroups (Table 7). In the first block, the post-test measures for the APSL scale (Model 1) were introduced, and the DS-EDPL measures were subsequently introduced into the analysis as an additional predictive factor (Model 2). Because the DS-EDPL significantly predicted the values for the dependent variables (attitude toward reading, and use of dialogue/participation strategies) after we controlled for the APSL scale scores, it can be concluded that the EDPL device provides additional information to that provided by the APSL scale.

**Table 6:** **Hierarchical multiple regression analysis for the entire group related to attitude toward reading, and use of dialogue/participation strategies in the classroom on the post-test for the APSL scale and the EDPL dynamic score**.

	**ATR-CT (*N* = 56)**					**DPS-CT (*N* = 56)**				
	**Beta**	***T (p)***		***R*^2^**	**Δ*R*^2^**	**Beta**	***T (p)***		***R*^2^**	**Δ*R*^2^**
Model 1				0.03	0.03				0.02	0.02
APSL	0.16	1.22	0.23			0.12	0.90	0.38		
Model 2				0.26	0.23^***^				0.29	0.27^***^
APSL	0.09	0.79	0.44			0.05	0.38	0.71		
DS-EDPL	0.49	4.05	<0.001			0.53	4.49	<0.001		

ATR-CT and DPS-CT, attitude toward reading, and use of dialogue/participation strategies in the classroom as evaluated by classroom teachers; ^*^p < 0.05; ^**^p < 0.01;

*** *p < 0.001 (a significant increase in the proportion of variance explained by model 2 is produced)*.

**Table 7:** **Hierarchical multiple regression analysis for the subgroups of students with intellectual disabilities, specific learning difficulties, and socio-cultural disadvantage, related to attitude toward reading, and use of dialogue/participation strategies in the classroom on the post-test for the APSL scale and the EDPL dynamic score**.

		**ATR-CT**					**DPS-CT**				
		**Beta(*n*)**	***T(p)***		***R*^2^**	**Δ*R*^2^**	**Beta(*n*)**	***T (p)***		***R*^2^**	**Δ*R*^2^**
Students with intellectual disabilities	Model 1				0.03	0.03				0.00	0.00
	APSL	0.17(18)	0.69	0.50			0.01(18)	0.05	0.97		
	Model 2				0.30	0.27^*^				0.35	0.35^**^
	APSL	0.04(18)	0.17	0.87			−0.14(18)	−0.66	0.52		
	DS-EDPL	0.53(18)	2.38	<0.05			0.61(18)	2.86	<0.01		
Students with specific learning difficulties	Model 1				0.04	0.04					
	APSL	0.21(24)	0.99	0.34			0.12(24)	0.57	0.57	0.02	0.02
	Model 2				0.56	0.51^***^				0.26	0.24^*^
	APSL	0.23(24)	1.59	0.13			0.14(24)	0.74	0.47		
	DS-EDPL	0.72(24)	4.92	<0.001			0.49(24)	2.61	<0.05		
Students with socio-cultural disadvantage	Model 1				0.02	0.02				0.05	0.05
	APSL	0.13(14)	0.45	0.66			0.22(14)	0.77	0.46		
	Model 2				0.10	0.09				0.36	0.31^*^
	APSL	−0.21(14)	−0.02	0.99			−0.04(14)	−0.15	0.88		
	DS-EDPL	0.33(14)	1.04	0.32			0.62(14)	2.34	<0.05		

ATR-CT and DPS-CT, attitude toward reading and use of dialogue/participation strategies in the classroom as evaluated by classroom teachers;

* p < 0.05;

** p < 0.01;

*** *p < 0.001 (a significant increase in the proportion of variance explained by model 2 is produced)*.

The results for the entire group show that including the DS-EDPL in the model significantly increased the predictive validity of the model with respect to the attitude toward reading, with an incremental validity of 23% (β = 0.49, *p* < 0.001). Such an increase was also observed for the use of dialogue/participation strategies in the classroom, with an incremental validity of 27% (β = 0.53, *p* < 0.001). Thus, the data support our second hypothesis, showing that including the DS-EDPL in the model significantly increased in the models' ability to predict students' attitudes toward reading and the use of dialogue/participation strategies.

Furthermore, using multiple regression analyses for the different subgroups (Table 7), we were able to assess the *differential validity* of the EDPL device according to the different characteristics present in the sample (Beckmann, 2006). Caffrey et al. (2008) have noted the importance of determining the different predictive validity of dynamic tests for the subgroup populations evaluated. For the ID subgroup (students with *intellectual disabilities* and no socio-cultural disadvantages), as for the entire group, the data revealed that the DS-EDPL had significant incremental validity in relation to the post-test measures obtained by the APSL scale. Such a significant increase was observed for students' attitudes toward reading and the use of dialogue/participation strategies in the classroom, with an incremental validity of 27% (β = 0.53, *p* < 0.05) and 35% (β = 0.61, *p* < 0.01), respectively.

Regarding the LD subgroup (students with *specific learning difficulties in reading* and no disabilities or socio-cultural disadvantages), the results of the analysis also revealed a significant increase in predictive validity. Specifically, the increase in predictive validity by the introduction of the DS-EDPL was 51% (β = 0.72, *p* < 0.001) for the assessment of students' attitudes toward reading. Regarding the assessment of the use of dialogue/participation strategies in the classroom, a significant increase in predictive validity of 24% (β = 0.49, *p* = 0.017) was observed.

Finally, regarding the SCD subgroup (students with *socio-cultural disadvantages* and with no disabilities), no significant increase in predictive validity was observed for the assessment of attitude toward reading. Regarding the assessment of the use of dialogue/participation strategies in the classroom, the inclusion of the DS-EDPL explained an additional 31% of the variance (β = 0.62, *p* = 0.039).

In short, the data obtained revealed some differences in predictive validity that depend on the subgroup studied. Consistent with our third hypothesis, more consistent values of incremental validity were obtained for the ID subgroup, and positive results were also obtained for the LD subgroup.

## Discussion

The present work aimed to establish the predictive and incremental validity of the EDPL device on students' attitudes toward reading, and the use of dialogue/participation strategies in the classroom during the application period. In our hypotheses, we predicted that dynamic scores on the EDPL would be significantly related to above-mentioned variables, and that the correlations would be higher for the EDPL than for the APSL scale. We also expected that the contextualized application of the EDPL device would provide additional information explaining students' attitudes toward reading, and the use of dialogue/participation strategies in relation to the information provided by the standard application of the APSL scale. In this sense, the results clearly reveal that the EDPL device showed a significant incremental validity with respect to the predictions based on the static test of personal-social adjustment.

The EDPL device and the APSL scale are clearly different assessment models. Although the EDPL device provide assessment indicators of personal-social adjustment processes that are similar to the items assessed in the APSL scale, the instruments differ essentially in the implementation modality as well as the punctuation format. In this way, the dynamic scores were obtained from the assessment and subsequent quantification by evaluators of the assessment indicators proposed in each activity. The valuation of these indicators was conducted for each of the activities through the system adopted. The predictive validity of the dynamic scores of the personal-social adjustment processes (DS-PSA) has specifically established with the 18 assessment indicators relative to this process (Table 2). For its part, the APSL scale score depends of the answers obtained in a Likert scale format. Assuming these differences, which limit the direct comparison between the results obtained, the fundamental objective of this study was, beyond comparison, to analyze the predictive validity and the additional information on the personal-social adjustment processes assessed, which could provide the implementation of the DA device.

In line with the above, although the control group was not analyzed in this article, some calculations were performed to assess the relevance of the implementation of the EDPL device. The data allowed us to establish a comparison with those obtained for the experimental group. First, in the control group the correlation level with academic performance as assessed by the classroom teachers was higher in the pre-test of APSL scale, which suggests that in this group the initial assessment would have revealed enough to report on the students' performances faithfully. Thus, in the absence of specific treatment, the initial prediction for performance would have been valid. However, for the experimental group the implementation of the EDPL device introduced a significant new element that would have supplanted the pre-test score in APSL scale as the strongest predictor. Indeed, both the global dynamic scores (*r* = 0.58; *p* < 0.01) and the dynamic scores of the personal-social adjustment processes, DS-PSA (*r* = 0.29; *p* < 0.05) correlated with academic performance as valued by classroom teachers significantly above the pre-test score (*r* = 0.19, *p* > 0.05).

In their review of DA models, Carlson and Wiedl (2000) noted the need for DA (a) to demonstrate a higher predictive validity than static tests with regard to the aptitude studied as well as to provide detailed knowledge of the effects of the model on factors related to aptitude (e.g., from aspects related to learning strategies to other aspects related to personal-social adjustment processes), and (b) to facilitate the connection between the mediation work aimed at general learning processes and strategies and the specific domains such as reading and comprehension tasks. Regarding the first point, our data show that the EDPL device has a higher predictive validity than the APSL scale and that it provides additional information on the significant effects of the model regarding the aspects studied (in this case, students' attitudes toward reading and the use of dialogue/participation strategies).

Regarding the second point highlighted by Carlson and Wiedl, the contextualized application of the EDPL device involves the constant need to connect and integrate the mediation work aimed at metacognitive learning strategies for the specific domain of reading and comprehension tasks. However, given that personal-social adjustment processes are closely related to school performance, which has been shown in numerous studies (Meltzer et al., 2004; Natale et al., 2009; Taboada et al., 2009), the activity developed during the mediation process that occurs during the application of the EDPL device involves a continuous and intentional educational intervention with respect to students' attitudes, motivation, self-interest, and approach to the learning situation. This intervention, which based on mediated interaction processes that account for the closeness and affection of the evaluator as integral elements, primarily aims to induce metacognitive reflection on processes that are related to the students' personal-social adjustment. Thus, metacognition would constitute a fundamental pillar of social and personal development (Hughes and Ensor, 2011), and difficulties in the metacognitive processes may constitute the basis not only of learning difficulties, but also of disorders related to personal-social adjustment (Mora-Merchán and Mora, 2000). In this sense, the contextualized integration (through reading tasks and in the regular educational context of the students) of the mediation work aimed at metacognitive and personal-social adjustment processes may account for the observed positive results.

Thus, the information obtained through the implementation of the EDPL device is qualitatively different from what can be obtained with the implementation of a standard test. This “additional” information deals mainly with difficulties manifested by the student during the process of solving the tasks as well as on those mediation guidelines that have proven effective in the implementation process. Further studies should develop a more precise analysis of this additional qualitative information, especially with the aim of identifying those mediation guidelines related specifically to an optimal response to students' interventions regarding the processes they have learned and practiced. It would then be possible to infer intervention procedures based on the contents and processes developed during the implementation of the device as well as the specific educational needs of the students.

In relation to our third hypothesis, the results of the differential validity analysis on the different subgroups are consistent with the results from previous research (Caffrey et al., 2008). In this sense, we can observe differences between the number and intensity of the additional predictive values of the three subgroups. Thus, the DS-EDPL has greater incremental validity with respect to the attitudes toward reading and the use of dialogue/participation strategies among the students in the ID and LD subgroups than for students in the SCD subgroup. Caffrey et al. (2008) have found that the predictive validity of dynamic tests was significantly higher and more consistent for students with intellectual disabilities than for students with socio-cultural disadvantages. In our study, we also observed an increase in the predictive validity for the LD subgroup, although to a lesser extent. The specific aspects that comprise personal-social adjustment processes (e.g., motivation, attitude, self-concept, expectations, or patterns of causal attribution) may have a more determinant role in the learning process for socio-culturally disadvantaged students. Such processes may interact with educational guidelines at school or in the family, with expectations and patterns of causal attribution of teachers and parents, and with difficulties related to the specific school context. These interactions may seriously hinder the effectiveness of programs aimed at improving students' cognitive skills, metacognition, and psychosocial adjustment (Mora, 1998). In our study, these interactions may have played an important role in the absence of significant improvements for socio-culturally disadvantaged students after the implementation of the EDPL device (Navarro and Mora, 2012), or in the lack of consistent predictive validity for the EDPL device with respect to performance in reading. The importance of such interactions suggests the need to incorporate other contextual elements (e.g., family and classroom teachers) in the programs and strategies aimed at improving students' reading performance. The obtained results regarding the differential predictive validity may nevertheless be particularly important for empirically determining the student populations that may most benefit from the application of the EDPL device in the school context.

As for the study's limitations, it is important to note the challenges arising from the characteristics of the study population, specifically with regard to the small sample size and the simultaneous evaluation of distinct subgroups. Although focusing on those subgroups can empirically help identify the population for which the device may provide more accurate information, it could also affect the conclusions as well as the potential for their generalization. It also important to consider the different evolutionary stages in relation to the processes evaluated. In this way, it must note that the teachers who participated in the study usually worked with small groups of students at different levels of development and competence and had to adapt the content and methods used continuously. The structure and sometimes the composition of these groups were the basis of the support groups in the implementation of EDPL.

Finally, although we previously indicated that the implementation of the device can provide additional qualitative information beyond that of static tests, it is necessary to take into account the specific training needs that entails. In practice, we cannot ignore that it is complicated to evaluate a learning process that involves (a) specific content relating to the tasks and student's learning difficulties, (b) mediation guidelines concerning aspects specific to both self-regulation and personal-social adjustment, and (c) indicators to assess student's achievements in each session. However, the study proved that after the initial training sessions, and, with more expertise, in later follow-up sessions, evaluators effectively implemented the mediation guidelines proposed and subsequently transferred their assessments to their registration-evaluation sheets. In their qualitative assessment of the strengths and weaknesses of the device, evaluators also highlighted that the indicators and mediation guidelines helped with observing difficulties and assessing the most effective assistance to offer the student. They also stressed the practical usefulness of the information obtained during the implementation process regarding the establishment of a constant connection between the processes of assessment and intervention.

## Author contributions

Conceived and designed the study: JJN. Performed tables and figures: JJN, LL. Analyzed the data: JJN, LL. Contributed materials/analysis tools: LL, JJN. Wrote the paper: JJN, LL.

### Conflict of interest statement

The authors declare that the research was conducted in the absence of any commercial or financial relationships that could be construed as a potential conflict of interest.
